# Educational needs and its associated factors among patients with ankylosing spondylitis in China: a multicenter cross-sectional study

**DOI:** 10.3389/fpubh.2024.1469863

**Published:** 2024-10-22

**Authors:** Yuqing Song, Weiping Shen, Xue Deng, Lu Xing, Yuping Tang, Mei Liu, Qiaolin Jiang, Yanling Chen, Benyi He, Li Wang, Fangmei Tang, Jianmei Wu

**Affiliations:** ^1^Department of Gynecological Nursing, West China Second University Hospital, Sichuan University, Chengdu, China; ^2^Key Laboratory of Birth Defects and Related Diseases of Women and Children (Sichuan University), Ministry of Education, Chengdu, China; ^3^Department of Rheumatology, Jiujiang No.1 People’s Hospital, Jiujiang, China; ^4^Department of Rheumatology, Second Hospital of Shanxi Medical University, Taiyuan, China; ^5^Rheumatology and Immunology Department, Deyang People’s Hospital, Deyang, China; ^6^Department of Rheumatology and Immunology, West China Hospital, Sichuan University, Chengdu, China; ^7^The First Hospital of Kunming, Kunming, China; ^8^Department of Rheumatology and Immunology, Hospital of Chengdu University of Traditional Chinese Medicine, Chengdu, China; ^9^Department of Obstetric Nursing, West China Second University Hospital, Chengdu, China

**Keywords:** educational needs, ankylosing spondylitis, patients, health problems, disease activity

## Abstract

**Objective:**

Patient education is an important part of ankylosing spondylitis (AS) management. Effective patient education should be targeted at specific priorities and needs of the patients. However, the educational needs of patients with AS in China have not been systematically explored. The purpose of this study was to assess the level of educational needs and analyze associated factors in patients with AS in China.

**Methods:**

This multicenter cross-sectional study was conducted at five hospitals in China. The Chinese version of the Educational Needs Assessment Tool (ENAT) was used to measure educational needs. Disease activity and physical function were assessed using the Bath Ankylosing Spondylitis Functional Index (BASFI) and Bath Ankylosing Spondylitis Disease Activity Index (BASDAI). Multiple linear regression analysis was used to identify the predictors of educational needs.

**Results:**

This study included 163 patients with AS. The mean ENAT score was 86.25 (31.64). Patients wanted to know more about the arthritis process, self-help measures, and treatments. Multiple linear regression analysis indicated that younger age, being female, higher disease activity, and no family history of AS (*p* < 0.05) were positive predictors of educational needs.

**Conclusion:**

Patients with AS in China have considerable educational needs, particularly in the domains of arthritis, self-help, and treatment. Female patients and patients with younger age, higher disease activity, and no family history may have higher educational needs. These factors should be considered when conducting need-based patient education programs. Healthcare professionals should integrate need-based patient education into rheumatology care in the future.

## 1 Introduction

Ankylosing spondylitis (AS) is one of the most common chronic inflammatory rheumatic diseases that often affects the sacroiliac joints and axial skeleton (1). The prevalence of AS in mainland China is 0.29% and continues to increase (2). AS affects men more than women, with a gender ratio of 2–3 to 1 (1, 3). Early-onset AS is characterized by symptoms that typically appear in the second or third decade of life. However, late-onset AS (LoAS) is estimated to occur in approximately 3.5–13.8% of all cases of AS (4, 5). AS often causes pain, joint damage, physical problems, inability to work, and impaired quality of life (6, 7). Moreover, the disease cannot be cured (8, 9). The main goal of treating patients with AS is to maintain health-related quality of life by controlling disease activity, preventing the progress of joint damage, and preserving function and social participation (8, 9). Thus, strategies should be developed to help patients manage their disease.

Patient education is an important component of AS management (9, 10). Patient education is an interactive process between patients and healthcare professionals that enables patients to manage their disease and improve coping strategies and quality of life (11). Previous evidence (10–12) suggested that tailored need-based education has positive effects on medication adherence and health outcomes. Meanwhile, the European League Against Rheumatism (EULAR) recommended that patient education should be tailored to address the individual needs and priorities for people suffering from inflammatory arthritis (10). Healthcare professionals should identify patients’ educational needs and priorities before developing effective and tailored patient education.

Existing studies that explore patients’ educational needs are mainly focused on patients with rheumatoid arthritis (RA) (13–18). The educational needs and priorities of AS patients have not been well-established. Haglund et al. (19) found that patients with spondyloarthritis (SpA), including AS, had considerable educational needs with important issues related to self-help, feelings, and disease processes. Marques et al. (20) found that patients with AS had a high level of educational needs in the “Disease process,” “Feelings,” and “Managing pain” domains. Several studies have reported that patients with AS have unmet needs (21–24). However, these studies cannot provide sufficient evidence for planning tailored education for AS patients. A patient-reported tool, the Educational Needs Assessment Tool (ENAT), was developed to help patients identify what they needed for patient education (25). The ENAT has been validated for systematically assessing the educational needs of different rheumatic diseases (including AS) and has been adapted into different languages (14, 26, 27). It is considered a straightforward and useful tool that can identify the needs of patients and direct healthcare professionals to develop patient education to meet patients’ needs (28, 29).

Thus, the educational needs among AS patients in China have not been systematically explored. This study aimed to assess the educational needs of Chinese patients with AS and analyze the associated factors. This will enable healthcare professionals to meet patient needs and enhance the effectiveness of patient education.

## 2 Materials and methods

### 2.1 Study design

This study used a multicenter cross-sectional design to systematically explore the educational needs of Chinese AS patients. This study was conducted at the Department of Rheumatology of five tertiary hospitals in China: Jiujiang No. 1 People’s Hospital, Second Hospital of Shanxi Medical University, Deyang People’s Hospital, Hospital of Chengdu University of Traditional Chinese Medicine, and West China Hospital, Sichuan University.

### 2.2 Participants

Potential patients were selected using convenience sampling from the Department of Rheumatology of five tertiary hospitals between July 2021 and November 2022. The inclusion criteria were as follows: (1) patients who met the Modified New York Classification Criteria for AS (30), (2) patients who were able to complete the questionnaire, (3) patients who had no mental impairment, and (4) patients who were 14 years and above. Patients who did not like to participate in this study and those with other concomitant rheumatic diseases and psychiatric disorders were excluded from the study.

### 2.3 Data collection and measures

#### 2.3.1 Demographic and disease-related data

Demographic data included age, gender, educational level, and ethnic group. Disease-related data included family history of AS (yes/no) and diagnosis duration. These data were collected using questionnaires from patients.

#### 2.3.2 Educational needs

Educational needs were assessed using the Chinese version of ENAT (31). In addition, the following two questions were used to obtain information on the overall educational needs of the patients: (1) whether they wanted education to help them deal with arthritis (yes/no) and (2) how much information they wanted to deal with arthritis, based on four answers, “I do not want to know anything,” “I want to know some things,” “I want to know lots of things,” and “I want to know everything.” The ENAT is a patient-reported questionnaire used to systematically identify the educational needs of patients with arthritis (25). The Chinese version of ENAT has 39 items and consists of eight domains: pain management (five items), movement (four items), feelings (four items), arthritis process (seven items), treatments (six items), self-help measures (six items), support systems (four items), and adjuvant therapy (three items) (31). Each item was scored on a 5-point Likert scale ranging from 0 (not at all important) to 4 (extremely important). Each domain score was calculated by summing all items from this domain. The total ENAT scores were calculated by summing all domain scores (0–156), with higher scores indicating higher demand for education (31). The standardized score of each domain was calculated to ensure that different domains could be compared (27, 32). The total scores for each domain were divided by the maximum possible domain score and then multiplied by 100 (25). Currently, the ENAT has proven to be a valid tool for estimating educational needs (14, 27). In the current study, Cronbach’s *α* for the total questionnaire was 0.969.

#### 2.3.3 Physical function

The Bath Ankylosing Spondylitis Functional Index (BASFI) was used to measure physical function in patients with AS. The BASFI is a 10-item self-completed instrument that reflects function and patients’ ability to deal with daily life (33). The total score ranges from 0 to 10 (33). A higher BASFI score indicates worse physical function.

#### 2.3.4 Disease activity

We used the Bath Ankylosing Spondylitis Disease Activity Index (BASDAI) to estimate the disease activity in patients with AS. The BASDAI is a self-report instrument consisting of six items to assess fatigue, spinal and peripheral joint pain, localized tenderness, and morning stiffness (34). The total BASDAI score ranges from 0 to 10, with higher scores indicating more severe disease activity.

### 2.4 Procedures and ethical considerations

The investigators at each hospital were trained before data collection. The investigators then used questionnaires to collect data from the participants when they visited the Department of Rheumatology for routine care. The study was approved by the West China Hospital (No. 20160364). It conformed to the Declaration of Helsinki to protect the human subjects. All participants were informed of the purpose and procedure of the study, and they provided written informed consent.

### 2.5 Statistical analysis

Statistical Package for the Social Sciences (SPSS 21.0, Chicago, IL, United States) was used to analyze the data. The normality of the data was evaluated using the Shapiro–Wilk test along with histograms, P–P plots, and Q-Q plots. Continuous data are presented as mean (standard deviation, SD) and median (interquartile range), and categorical data are presented as frequencies and percentages (%). Spearman’s correlation analysis was used to explore the relationships between educational needs and age, disease duration, BASFI score, and BASDAI score. The closer the r-value comes to 1, the stronger the relationship (35). The strength of the correlation was classified as negligible (0 ≤ r < 0.3), weak (0.30 ≤ r < 0.50), moderate (0.5 ≤ r < 0.7), and strong (0.70 ≤ r ≤ 1) (35). An independent-samples *t*-test was used to compare the educational needs of the patient subgroups. Finally, predictors of educational needs were determined using multiple linear regression analysis. Statistical significance was set at a *p*-value of <0.05.

## 3 Results

### 3.1 Characteristics of participants

A total of 180 patients completed the questionnaires. However, we excluded 17 patients due to incomplete questionnaires and included 163 patients with AS in this study (135 male patients and 28 female patients). The mean (SD) age of AS patients was 33.18 (10.02) years, ranging from 14 to 70 years. The median year of disease duration was 4.00. Most of the participants were Han people and reported no family history of AS. The median BASDAI and BASFI scores were 3.20 and 1.30, respectively. The characteristics of 163 patients with AS are presented in Table 1.

**Table 1:** Characteristics of patients with ankylosing spondylitis.

Variables	Mean (SD)/median (IQR)	Frequency (%)	Mean (% of maximum)
Age, mean (SD)	33.18 (10.02)		
Disease duration (year), median (IQR)	4.00 (8.00)		
Gender, *n* (%)			
Female		28 (17.2)	
Male		135 (82.8)	
Ethnic group, *n* (%)			
Han nationality		144 (88.3)	
Ethnic minorities		19 (11.7)	
Education level			
Middle school and below		84 (51.5)	
College and above		79 (48.5)	
Family history of AS			
Yes		34 (20.9)	
No		129 (79.1)	
BASDAI score, median (IQR)	3.20 (3.20)		
BASFI score, median (IQR)	1.30 (3.00)		

SD, standard deviation; IQR, interquartile range; N, number; AS, ankylosing spondylitis; BASFI, Bath Ankylosing Spondylitis Functional Index; BASDAI, Bath Ankylosing Spondylitis Disease Activity Index.

### 3.2 Educational needs

The educational needs of AS patients are presented in Table 2. In the current study, most of the participants (*n* = 131, 80.4%) wanted an educational intervention to help them deal with AS, and 38.7% (*n* = 63) of the patients would like to know ‘everything to deal with AS’. The mean ENAT score was 86.25 (31.64). The highest domain scores for educational needs were found for the arthritis process (18.12 ± 7.01), self-help measures (14.71 ± 6.12), and treatments (15.10 ± 5.78).

**Table 2:** Educational needs of patients with ankylosing spondylitis.

Variables	Mean (SD)/median (IQR)	Frequency (%)	Mean (% of maximum)
Do you want education?			
Yes		131 (80.4)	
No		32 (19.6)	
How much information do you want?			
I do not want to know anything		5 (3.1)	
I want to know some things		21 (12.9)	
I want to know lots of things		74 (45.4)	
I want to know everything		63 (38.7)	
Educational needs			
Managing pain (0–20)	7.52 (4.64)		37.6%
Movement (0–16)	8.27 (3.69)		51.7%
Feelings (0–16)	7.80 (4.39)		48.8%
Arthritis process (0–28)	18.12 (7.01)		64.7%
Treatments (0–24)	15.10 (5.78)		62.9%
Self-help measures (0–24)	14.71 (6.12)		61.3%
Support systems (0–16)	9.21 (4.38)		57.6%
Adjuvant therapy (0–12)	5.51 (3.23)		45.9%
Total score (0–156)	86.25 (31.64)		55.3%

### 3.3 Variables associated with educational needs measured by ENAT

Table 3 presents the relationships between age, disease duration, BASDAI scores, and BASFI scores, and educational needs of patients with AS. Older age was correlated with lower educational needs in the movement domain (r = −0.190, *p* = 0.015). Longer disease duration was associated with higher educational needs in the domain of support system (r = 0.180, *p* = 0.022). Higher disease activity was associated with higher domain scores for feelings (r = 0.194, *p* = 0.013), arthritis process (r = 0.180, *p* = 0.021), treatments (r = 0.155, *p* = 0.049), support systems (r = 0.192, *p* = 0.014), adjuvant therapy (r = 0.205, *p* = 0.009), and total scores (r = 0.172, *p* = 0.028). The results indicated negligible correlation between disease duration, age, disease activity, and educational needs. However, no statistical correlation was detected between physical function and educational needs in our study.

**Table 3:** Correlation of educational needs with age, disease duration, BASDAI, and BASFI.

Variables	Age		Disease duration		BASDAI		BASFI	
	r	*p*	r	*p*	r	*p*	r	*p*
Managing pain	−0.048	0.546	−0.040	0.615	0.067	0.395	0.005	0.954
Movement	−0.190	0.015*	−0.036	0.646	0.063	0.427	−0.066	0.403
Feelings	−0.049	0.533	0.043	0.586	0.194	0.013*	0.057	0.470
Arthritis process	−0.152	0.053	0.087	0.269	0.180	0.021*	0.089	0.258
Treatments	−0.104	0.186	0.095	0.229	0.155	0.049*	0.040	0.611
Self-help measures	−0.067	0.394	0.072	0.358	0.083	0.292	−0.055	0.482
Support systems	−0.128	0.104	0.180	0.022*	0.192	0.014*	0.067	0.394
Adjuvant therapy	−0.088	0.264	−0.029	0.709	0.205	0.009*	0.148	0.059
Total score	−0.153	0.052	0.073	0.357	0.172	0.028*	0.013	0.697

BASFI, Bath Ankylosing Spondylitis Functional Index; BASDAI, Bath Ankylosing Spondylitis Disease Activity Index. *p < 0.05.

Table 4 summarizes the differences in educational needs according to gender, ethnic group, family history of AS, and educational level. Female patients had significantly higher educational needs regarding the arthritis process and self-help measures than male patients (*p* < 0.05). Han people scored higher on educational needs within the domains of feelings, arthritis process, and support systems (*p* < 0.05). Patients with a family history of AS reported lower educational needs regarding the arthritis process, treatments, support systems, adjuvant therapy, and total scores (all *p* < 0.05). There were no statistically significant differences in the ENAT scores between patients with different educational levels.

**Table 4:** Difference in AS patients’ educational needs by gender, ethnic group, family history, and educational background.

Variables	Gender				Ethnic group			
	Male (*n* = 135)	Female (*n* = 28)	t^a^	*p*	Han (n = 144)	Other (n = 19)	t^a^	*p*
Managing pain	7.59 (4.90)	7.21 (3.12)	0.512	0.611	7.57 (4.74)	7.16 (3.83)	0.363	0.717
Movement	8.27 (3.82)	8.29 (3.09)	−0.025	0.980	8.35 (3.72)	7.68 (3.51)	0.734	0.464
Feelings	7.55 (4.54)	9.04 (3.36)	−1.641	0.103	8.16 (4.38)	5.11 (3.45)	2.918	0.004*
Arthritis process	17.64 (7.21)	20.43 (5.47)	−2.316	0.025*	18.51 (6.98)	15.11 (6.70)	2.011	0.046*
Treatments	14.74 (5.98)	16.86 (4.37)	−1.776	0.078	15.33 (5.79)	13.42 (5.54)	1.355	0.177
Self-help measures	14.16 (6.28)	17.36 (4.56)	−2.555	0.012*	14.98 (6.15)	12.68 (5.68)	1.542	0.125
Support systems	8.93 (4.42)	10.61 (3.95)	−1.863	0.064	9.45(4.48)	7.42 (3.02)	2.577	0.015*
Adjuvant therapy	5.41 (3.26)	6.00 (3.08)	−0.883	0.378	5.61 (3.31)	4.74 (2.51)	1.110	0.269
Total score	84.27 (32.93)	95.79 (22.60)	−1.764	0.080	87.95 (32.09)	73.32 (25.01)	1.912	0.058

Variables	Family history of AS				Education level (middle school and below)			
	No (*n* = 129)	Yes (*n* = 34)	t^a^	*p*-value	Yes (*n* = 84)	No (*n* = 79)	t^a^	*p*-value
Managing pain	7.53 (4.78)	7.50 (4.08)	0.030	0.976	7.83 (4.99)	7.19 (4.23)	0.885	0.377
Movement	8.33 (3.72)	8.03 (3.62)	0.426	0.671	7.98 (3.73)	8.58 (3.65)	−1.047	0.297
Feelings	8.10 (4.31)	6.67 (4.57)	1.694	0.092	7.69 (4.36)	7.92 (4.44)	−0.339	0.735
Arthritis process	18.85 (6.61)	15.32 (7.86)	2.660	0.009*	17.90 (6.97)	18.34 (7.09)	−0.397	0.692
Treatments	15.67 (5.43)	12.94 (6.59)	2.493	0.014*	14.98(5.90)	15.24 (5.68)	−0.291	0.771
Self-help measures	15.04 (6.13)	13.47 (6.02)	1.332	0.185	14.25 (6.26)	15.20 (5.97)	−0.993	0.322
Support systems	9.65 (4.32)	7.56 (4.27)	2.520	0.013*	9.25 (4.60)	9.17 (4.16)	0.106	0.916
Adjuvant therapy	5.81 (3.19)	4.38 (3.17)	2.318	0.022*	5.42 (3.32)	5.61 (3.15)	−0.376	0.707
Total score	88.98 (31.18)	75.88 (31.66)	2.173	0.031*	85.30 (32.88)	87.27 (30.43)	−0.396	0.693

AS, ankylosing spondylitis. *p < 0.05.

a Independent-samples *t*-test.

Table 5 describes the results of the multiple linear regression analysis using ENAT total scores as the dependent variable. Younger age (*p* = 0.004), higher disease activity (*p* = 0.007), being female (*p* = 0.035), and no family history of AS (*p* = 0.043) were significant predictors of higher educational needs. These variables accounted for 11.6% of the variance in the ENAT total scores.

**Table 5:** Multiple linear regression analysis.

Variable	B	Standardized coefficients (β)	t	*p*
Age	−0.767	−0.243	−2.938	0.004*
BASDAI	3.990	0.279	2.744	0.007*
BASFI	−2.080	−0.165	−1.602	0.111
Disease duration	0.665	0.125	1.524	0.130
Gender (reference: Male)				
Female	13.717	0.164	2.127	0.035*
Ethnic group (reference: ethnic minorities)				
Han nationality	13.992	0.142	1.881	0.062
Family history of AS (reference: no)				
Yes	−11.943	−0.154	−2.045	0.043*
Education level (reference: middle school and below)				
College and above	1.557	0.025	0.319	0.750

AS, ankylosing spondylitis; BASDAI, Bath Ankylosing Spondylitis Disease Activity Index; BASFI, Bath Ankylosing Spondylitis Function Index. *p < 0.05.

## 4 Discussion

This study used the ENAT to explore the educational needs of Chinese patients with AS and analyzed its predictors. We found that approximately 80.4% of patients expressed a need for education to better manage their disease. The mean (SD) ENAT total score was 86.25 (31.64), which is similar to previous studies on spondyloarthritis patients (19, 36), systemic lupus erythematosus (SLE) patients (37), and AS patients (20), but higher than those of systemic sclerosis, rheumatoid arthritis, and systemic vasculitis (32, 38–40). Patients reported that the most important educational needs were found in the domains of the arthritis process, self-help measures, and treatments. These results were partially similar to a study in Portugal: Patients with AS had higher educational needs in the disease process, self-help measures, and managing pain domains (20). In addition, previous studies have revealed that patients with RA and SpA have high educational needs regarding treatment, self-help measures, and arthritis process (16, 19). This study also demonstrated that Chinese patients with AS had considerable educational needs. The ENAT is a simple and useful tool in both research and clinical practice (28). In the future, ENAT could be used to identify educational needs and develop education. Tailored patient education focusing on disease and medical care should be integrated into standard rheumatology care.

In the current study, younger patients with AS were found to have higher educational needs. Since some patients develop and are diagnosed with AS before 18 years (41), it is also important to know their needs. We did not exclude teenagers, which may have resulted in a higher proportion of younger patients included in our study. Previous studies on patients with RA have indicated that younger patients have a greater need for information and education (13, 38). Inflammatory arthritis, particularly AS, typically affects young people at their work age (42). Meanwhile, younger patients might have a deficit in knowledge and strategies to cope with the disease and life well (16). This may result in a greater need for information and education to manage the disease in younger patients. Thus, established patient education programs should focus on younger patients with AS.

We found that female patients had higher educational needs than male patients. An earlier study on patients with SpA and AS revealed that female patients expressed higher educational needs in the domains of pain management, movement, feelings, self-help, and total scores compared with male patients (19, 20, 36). Similarly, Cooksey et al. (22) reported that women were more likely to seek information about AS than men. Compared to male patients, female patients with AS had a higher disease burden and less improvement in outcome measures when receiving treatment (43). Patients with severe health problems may seek medical information (44). In addition, men with arthritis prioritize their work commitments over health problems, which may result in less need for patient education for male patients (44, 45). Therefore, gender differences should be considered before conducting tailored education for patients with AS.

Undoubtedly, patients with more active disease have a higher demand for education than patients with rheumatic disease (19, 36, 37, 39, 44). We found that disease severity was significantly correlated with higher educational needs in AS patients. Patients may ask for information and education to cope with the disease and their life when the disease affects them (44). Sierakowska et al. (39) revealed that patients with severe disease are more likely to benefit from patient education. In future studies and clinical practice, health professionals can use ENAT to identify patients with the highest educational needs and patients who are most interested in education to provide the most effective patient education.

In addition, patients with a family history of AS had a lower need for education than those with no family history of AS. A possible reason may be that patients with family history can discuss and receive information about AS from their families, which may result in relatively lower educational needs for these patients. Healthcare professionals should provide adequate educational information and opportunities for communicating with other patients to AS patients, especially those without a family history.

Although several studies have shown that disease duration, functional limitation, and educational level are associated with educational needs in systemic sclerosis and RA (38, 39), no significant association was detected in the current study. Haglund et al. (19) found that disease duration did not affect the educational needs of patients with SpA. Schouffoer et al. (32) revealed no association between educational needs and the duration of disease or educational level in systemic sclerosis. Further studies should explore the effects of these variables on the educational needs of patients with AS.

## 5 Limitations and future directions

The current study had several limitations. Although we recruited participants from five hospitals in China, all participants were recruited using convenience sampling, which may have limited the results of this study. As the ENAT has proven to be a valid questionnaire for assessing the educational needs of patients with arthritis, the tool has only seven domains and does not cover all information on educational needs in patients with AS. Future studies should use a mixed method to explore the educational needs of AS patients. The older adults has been increasingly globally. The treatment approach in geriatric rheumatological patients is similar to that in adult patients. As life expectancy increases, the incidence of rheumatological diseases in the older adults also increases. Therefore, studies that include older age groups are needed. Finally, this cross-sectional study did not detect fluctuations in educational needs. Future studies should use a longitudinal research design to explore the fluctuations in educational needs.

## 6 Conclusion

Patients had considerable educational needs in China, and the arthritis process, self-help measures, and treatment have been identified as the most important domains. In addition, younger age, being female, higher disease activity, and no family history of AS would lead to a greater need for education. These factors should be considered when providing effective education to patients. Our findings suggest that need-based patient education should be integrated into rheumatology care.

## Data Availability

The original contributions presented in the study are included in the article, further inquiries can be directed to the corresponding author.
